# Adipose-derived mesenchymal stem cells for the prevention of capsular contracture

**DOI:** 10.1007/s00109-025-02576-3

**Published:** 2025-08-01

**Authors:** Orel Govrin-Yehudain, Yasmin Korzets, Yosef Zkika, Noam Castel, Rafael Y. Brzezinski, Debby Reuveni, Yoad Govrin-Yehudain, Eyal Gur, Inna Solodeev, Yoav Barnea

**Affiliations:** 1 Department of Plastic and Reconstructive Surgery, Tel Aviv Sourasky Medical Center, Tel Aviv, Israel; 2 Gray Faculty of Medical and Health Sciences, Tel Aviv University, Tel Aviv, Israel; 3 Institute of Radiation Therapy, Tel Aviv Sourasky Medical Center, Tel Aviv, Israel; 4 Institute of Pathology, Tel Aviv Sourasky Medical Center, Tel Aviv, Israel; 5 Department of Plastic and Reconstructive Surgery, Meir Medical Center, Kfar Saba, Israel; 6 Research Center for Liver Diseases, Sheba Medical Center, Ramat Gan, Israel; 7 Department of Plastic and Reconstructive Surgery, Shamir Medical Center, Zerifin, Israel

**Keywords:** Capsular contracture, Radiation, Adipose-derived mesenchymal stem cells

## Abstract

**Abstract:**

Capsular contracture (CC) is the most common complication associated with implant-based breast surgery, with particularly high risk in patients undergoing alloplastic reconstruction surgery followed by radiation therapy. Revision surgery, the only currently effective treatment, carries a high risk of recurrent CC and secondary complications. This work assessed the prophylactic potential of human adipose-derived mesenchymal stem cells (hAD-MSCs) in a novel animal model of radiation-induced CC. A total of 36 female C57Bl/6 mice were randomly assigned to three groups: (1) IMP (silicone implants only), (2) IMP + RAD (silicone implants and irradiation therapy to promote CC), and (3) IMP + RAD + MSCs (silicone implants, irradiation therapy, and local administration of hAD-MSCs). On day 42 post-implantation, animals were euthanized and capsular tissue was subjected to histological and gene expression analyses. In addition, serum transforming growth factor beta (TGF-β) levels were measured. Targeted radiotherapy induced significant CC. In contrast, on day 42 post-irradiation, the capsular thickness in the IMP + RAD + MSCs group was significantly lower, comparable to that of non-irradiated mice. hAD-MSC treatment also resulted in a significant downregulation of pro-fibrotic and pro-inflammatory genes in the capsular tissue. In this study conducted in a murine model, hAD-MSCs demonstrated significant prophylactic potential in preventing radiation-induced CC. Further research is necessary to investigate the underlying mechanisms and to assess the efficacy and safety of this approach.

**Key messages:**

-  Radiotherapy increased capsular thickness and expression of pro-inflammatory and fibrotic genes.
-  Stem cells restored capsular thickness to levels comparable to non-irradiated mice.
-  Stem cells downregulated both pro-fibrotic and pro-inflammatory genes in capsule tissue.
-  Findings highlight the potential of stem cells in preventing radiation-induced capsular contracture.

## Background

Capsular contracture (CC) is the most common complication of implant-based aesthetic and reconstructive breast surgery [1–5]. While the incidence of CC following primary breast augmentation surgery ranges from 0.57 to 15%, [1, 2, 6, 7] the risk is higher in patients undergoing implant-based breast reconstruction (5–55%) [1, 8–12] and even higher in patients who undergo postmastectomy radiation therapy (PMRT). A recent meta-analysis reported CC rates of up to 50% in this high-risk group, [13] while other authors report rates of between 68 and 100% [1, 3, 4, 14, 15].

CC is thought to occur due to an excessive fibrotic reaction to the implant, resulting in excessively firm, painful, and deformed breasts [4–6]. Notably, thin capsule formation is a normal response to foreign bodies, whereas CC is not [5]. Numerous experimental and clinical studies have attempted to understand the risk factors, clinical features, and management of CC. The underlying etiology and optimal treatment of CC remain elusive [16, 17]. Feasible etiological factors include biofilm, hematoma, implant type, silicone leakage, and individual susceptibility [1]. Proposed risk factors for CC include incision site, implant surface texture, and radiotherapy [18].

The extensively documented correlation between radiation and CC has been ascribed to radiotherapy-induced tissue damage, which leads to increased inflammation, fibrosis and collagen deposition, ultimately contributing to the formation of a contracted capsule. Transforming growth factor beta (TGF-ß) has been identified as a pivotal cytokine involved in the development of radiation-induced fibrosis [3], through its stimulation of the production of extracellular matrix, leading to capsular fibrosis and contracture [19].

Thus far, no interventions have satisfactorily prevented or treated CC [20–26]. Currently, the only effective treatment for CC is surgery, which includes capsulotomy or capsulectomy with implant removal or replacement. Yet, the procedure carries a high risk for CC recurrence and secondary complications [1, 27].

In recent years, stem cells, particularly adipose-derived stem cells, have attracted considerable attention as promising candidates for anti-fibrosis treatment and regenerative medicine [28–30]. Stem cells can be isolated from adipose tissue with less invasive techniques and at higher yields as compared to bone marrow aspirates, and have significant proliferative capacity and a longer life span in culture [29]. Their anti-fibrotic, immunomodulatory, and angiogenic effects, among others, render them unique and suitable for treating a variety of fibrosis-related diseases [30, 31]. To the best of our knowledge, no studies have examined the prophylactic potential of human adipose-derived mesenchymal stem cells (hAD-MSCs) for the prevention of radiation-induced CC.

To date, most animal experimental models in this realm have been exploited to evaluate the outcome of different interventions on normal capsule formation and have failed to accurately replicate the development of CC [32–34]. Adams et al. were one of the first to study a pathological CC animal model that mimicked the formation of the CC response in humans [35]. Their study demonstrated a correlation between CC severity and the thickness of the capsule surrounding a saline implant. However, it was limited by the use of fibrin glue to induce CC, which does not replicate clinical reality. Katzel et al. were the first to study the effect of radiation on CC induction in a mouse model [36]. Radiation was shown to cause consistent reproducible changes with micro-computed-tomography and histology. The model utilized gamma radiation, similar to the radiation used in therapeutic breast cancer treatment, making it more representative of real-life conditions [13].

The current work assessed the potential of locally administered hAD-MSCs in the prevention of radiation-induced CC in mice.

## Materials and methods

### Study design

This study was approved by the Sourasky Medical Center—Institutional Animal Care and Use Committee, Israel. Eight-week-old, female C57Bl/6 mice, weighing 20 g, on average, were randomly assigned to three groups (12 mice per group): (1) IMP (silicone implants only), (2) IMP + RAD (silicone implants and irradiation therapy), and (3) IMP + RAD + MSCs (silicone implants, irradiation therapy, and local administration of hAD-MSCs). All mice underwent an acclimatization week upon arrival. On day 0, they were weighed and marked.

### Human adipose-derived mesenchymal stem cell isolation

Adipose tissue samples were obtained from patients undergoing liposuction surgery. All procedures were performed in accordance with the Declaration of Helsinki and approved by the ethics committee of Tel Aviv Sourasky Medical Center, Israel (approval no. 0369–12-TLV). Signed informed consent was obtained from patients before surgery. Cells were isolated from lipoaspirates using 0.1% collagenase (Sigma, St. Louis, MO, USA) and separated from the fat by centrifugation (15 min, 400 × g). hAD-MSCs were grown in high-glucose Dulbecco’s modified Eagle’s medium (DMEM) (Gibco, Paisley, Scotland, UK), supplemented with 10% fetal calf serum (Thermo Scientific HyClone, Tauranaga, New Zealand), 60 μg/ml penicillin, 100 μg/ml streptomycin, 50 μg/ml kanamycin, 1 mM sodium pyruvate, 2 mM L-glutamine, and non-essential amino acids, under 10% CO_2_ and atmospheric oxygen conditions. After 72 h, nonadherent cells were removed, while adherent cells were grown for 13 days.

Flow cytometry was performed to confirm that the cultured hAD-MSCs express stem cell markers. Cells were harvested and incubated (1 h, in the dark) with a seven-color panel containing anti-CD31, anti-CD34, anti-CD29, anti-CD105, anti-human-73, and anti-CD45 antibodies. To exclude dead cells, the samples were stained with ViViD (violet viability dye, Molecular Probes, Invitrogen, Eugene, OR, USA) according to the manufacturer’s protocol. Appropriate single-stained and isotype control samples were prepared and analyzed by flow cytometry (FACSCanto II, BD Biosciences). Flow-Jo software (Tree star, Ashland, OR, USA) was used for data analysis.

### In-vivo model

Mice were anesthetized with an intraperitoneal injection of ketamine (2 mg/0.47 mg) and xylazine (200 µl/mouse). Following anesthesia, the surgical site was shaved and prepared with iodine solution. A 1.5-cm transverse incision was made on the dorsal aspect of the mouse at the level of the sacral spine, followed by dissection of a cranial pocket, and subcutaneous placement of a smooth silicone mini-implant device (Polytech Health and Aesthetics, Germany). The incision was closed with interrupted 4–0 nylon sutures (Ethicon, Inc., Somerville, NJ, USA).

Following surgery and while still under anesthesia, animals in the radiation arms (IMP + RAD and IMP + RAD + MSCs) received a 10 Gy dose, delivered with a linear accelerator with 6 MeV x-ray beam (Varian True Beam). The radiation was targeted to induce CC around the silicone implants while avoiding systemic effects of radiation.

Following surgery and radiation, 3 × 10^6^ harvested hAD-MSCs suspended in 400 μL phosphate-buffered saline were locally administered by microinjection into the breast implant pocket of animals in the therapeutic group (IMP + RAD + MSCs).

Follow-up included daily evaluations for 5 consecutive days, and then once every 3 days. Each evaluation included assessment of general well-being and signs of distress, a physical examination, and weight measurement.

On postoperative day 42, total capsulectomy was performed and the capsules were sent for histological analysis. Gene expression was analyzed by real-time polymerase chain reaction (PCR). Intracardiac blood was collected to determine serum transforming growth factor beta (TGF-β) levels by enzyme-linked immunosorbent assay (ELISA). Mice were then euthanized by CO2 asphyxiation followed by cervical dislocation. The work has been reported in line with the ARRIVE guidelines 2.0.

### Histopathological examination

Capsules were harvested from all mice on postoperative day 42. Harvested tissues were fixed in 10% neutral buffered formalin and then embedded in paraffin blocks. Serial longitudinal sections were prepared and stained with hematoxylin and eosin for histological analysis of capsular thickness. All slides were scanned and capsular thicknesses in the three thickest and the three thinnest areas of the capsules were measured by a blinded pathologist.

### Quantitative real-time PCR

RNA was isolated from capsules with Tri-Reagent (Sigma, USA) and reverse-transcribed with the High-Capacity cDNA Reversed transcription kit (Applied Biosystems, USA) according to the manufacturer’s instructions. PCRs were performed with the SYBER Green PCR Master Mix (Applied Biosystems, USA). Quantification was performed using Step One software (V2.2). Levels of TGF-β, collagen type 1 (Coll1), alpha-smooth muscle actin (αSMA), tissue inhibitor of metalloproteinase 1 (TIMP1), interleukin-1 beta (IL-1β), and tumor necrosis factor alpha (TNFα) gene expression were compared with those of the glyceraldehyde 3-phosphate dehydrogenase (GAPDH) housekeeping gene. The primer sequences (forward and reverse) are listed in Table 1.

**Table 1:** Primer sequences used for quantitative real-time polymerase chain reaction (PCR)

Gene	Forward	Reverse
Coll-1	5′-GAGAGCATGACCGATGGATT-3′	5′-CCTTCTTGAGGTTGCCAGTC-3′
TGFβ	5′-ATTCAGCGCTCACTGCTCTT-3′	5′-GTTGGTATCCAGGGCTCTCC-3′
Timp-1	5′-TCCCCAGAAATCAACGAGAC-3′	5′-CTGGGACTTGTGGGCATATC-3′
αSMA	5′-CCCCTGAAGAGCATCGGACA-3′	5′-TGGCGGGGACATTGAAGGT-3′
TNFα	5′-CGAGTGACAAGCCTGTAGCC-3′	5′-CCTTGTCCCTTGAAGAGAACC-3′
IL-1β	5′-GACCTTCCAGGATGAGGACA-3′	5′-AGCTCATATGGGTCCGACAG-3′
GAPDH	5′-AACGACCCCTTCATTGAC-3′	5′-TCCACGACATACTCAGCAC-3′

### Enzyme-linked immunosorbent assay (ELISA)

Intracardial serum levels of TGF-β were measured using the TGF beta-1/LAP Mouse Uncoated ELISA Kit (INVITROGEN, USA) according to the manufacturer’s protocol.

### Sample size calculation

Sample size was calculated using G-power software with the following assumptions: type 1 error of 5% and minimum desired power of 80%. The primary endpoint to determine the sample size is the reduction in the capsular thickness and formation following the treatment with stem cells. Following a similar study conducted by Sutthiwanjampa et al. (2021), we assume a moderate effect size of this reduction. Specifically, using a mixed-model ANOVA, we examine the interaction between time and group (Time X Group), that is different pattern of changes between the four groups. Under these assumptions, the required sample size is 12 subjects in each group.
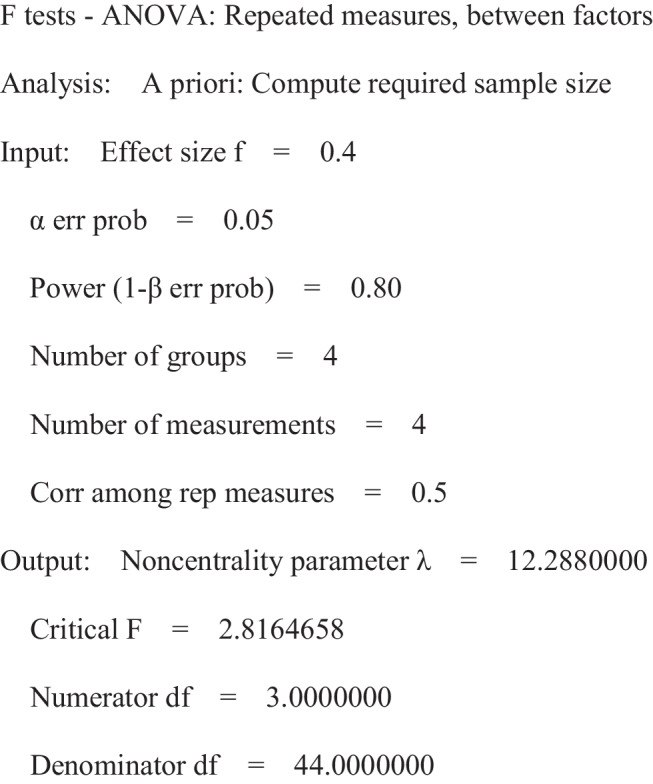


The required sample size is 12 subjects in each group. Total number of animals for the study: 36 mice.

### Statistical analysis

Variables are expressed as mean ± standard error of the mean (SEM). Differences between values were tested by one-way analysis of variance followed by Tukey’s test for multiple comparisons. If values were not normally distributed (determined by the D’Agostino Pearson omnibus normality test), the non-parametric Kruskal–Wallis test was used. *p* values of < 0.05 were considered statistically significant. All statistical analyses were performed with GraphPad Prism version 9.00 (GraphPad Software, La Jolla, CA, USA).

## Results

### Targeted radiation-induced capsular contracture in mice

**Fig. 1:**
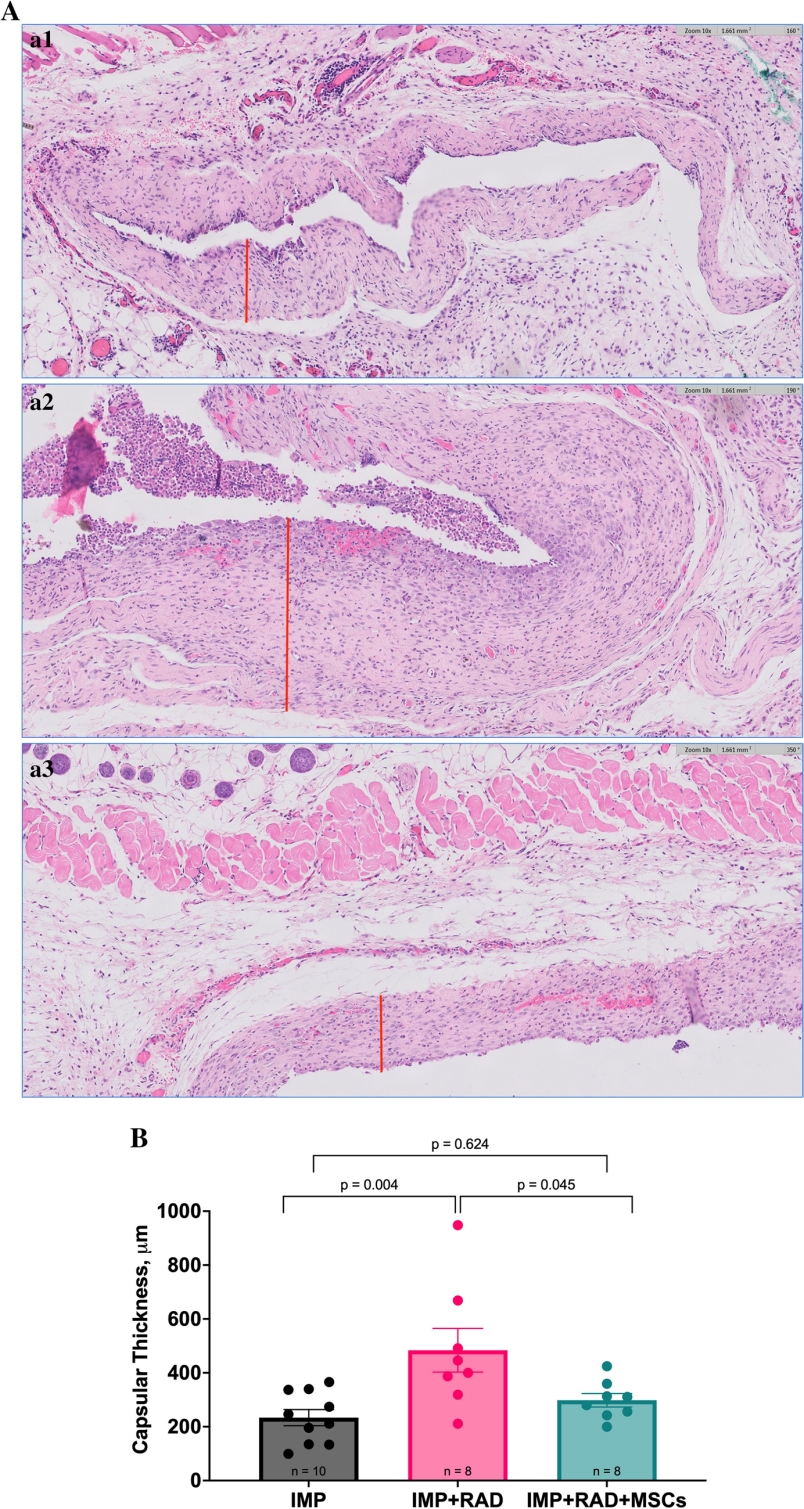
Capsular thickness assessed by histopathology. **A** Representative histological sections of capsules extracted on postoperative day 42 from a mouse with (**A1**) a silicone implant only (IMP), (**A2**) a silicone implant and then subjected to radiation therapy (IMP + RAD), or (**A3**) a silicone implant, and then subjected to radiation therapy and localized administration of human adipose-derived mesenchymal stem cells (IMP + RAD + MSCs). Sections were stained with hematoxylin and eosin (magnification × 10). The red line marks capsular thickness.** B** Radiotherapy significantly increased the mean capsular thickness as measured by histopathological examination 42 days following implant insertion. Human adipose-derived mesenchymal stem cell (hAD-MSC) treatment significantly reduced the mean capsular thickness to levels similar to those seen in mice with an implant but who were not subjected to radiation. Data are shown as the mean ± SEM. *p* values were calculated by a one-way analysis of variance followed by Tukey’s test for multiple comparisons

**Fig. 2:**
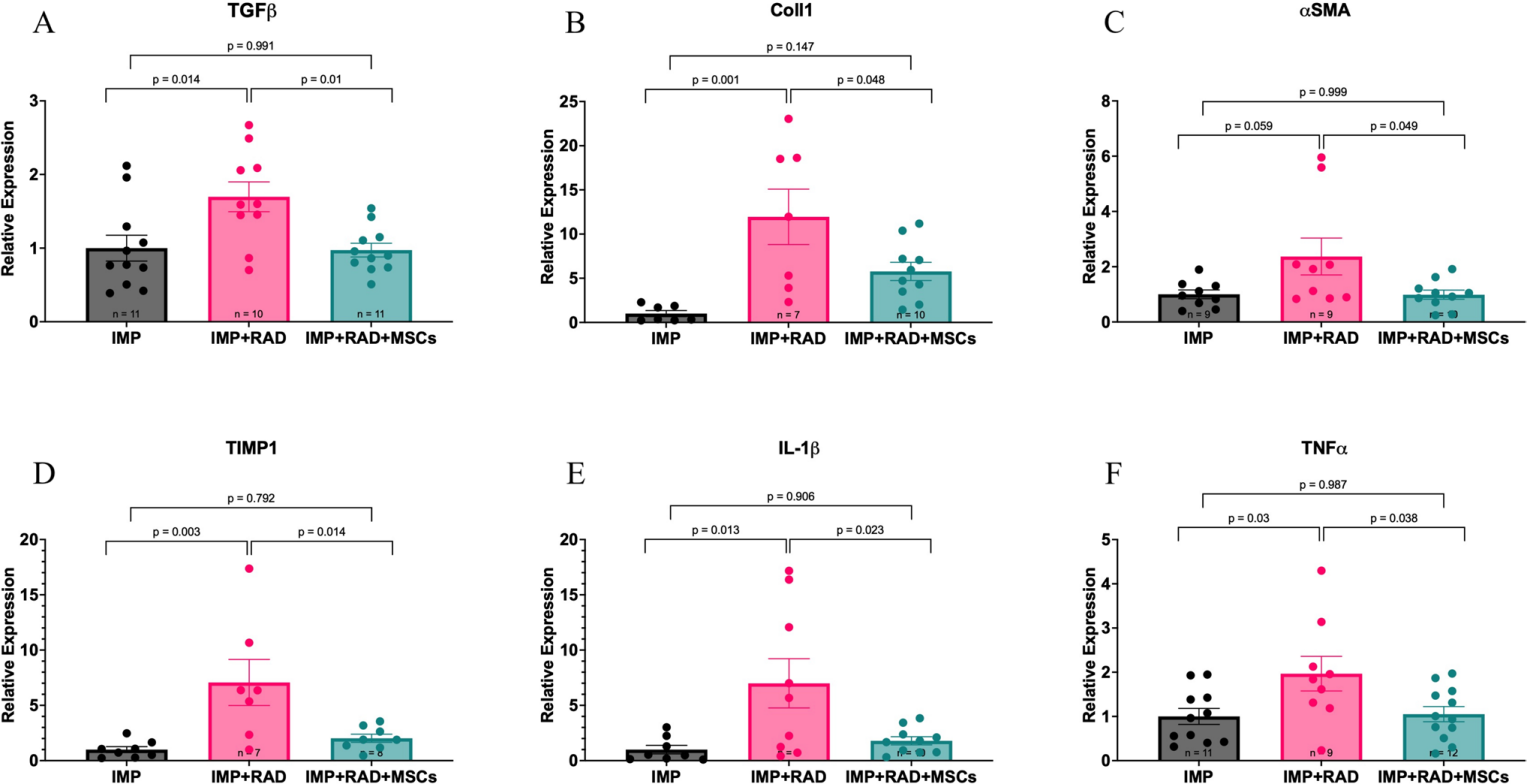
Gene expression in capsular tissues isolated 42 days post-implantation. Radiotherapy significantly upregulated the expression of pro-fibrotic and pro-inflammatory genes in the capsular tissue. The administration of human adipose-derived mesenchymal stem cells significantly downregulated the levels of the pro-fibrotic genes TGF-β (**A**), collagen1 (**B**), α-SMA (**C**), and TIMP1 (**D**) as well as the pro-inflammatory genes IL-1β (**E**) and TNFα (**F**). Quantitative RT-PCR 2 − ΔΔCT values were calculated using GAPDH as the housekeeping gene. Data are shown as the mean ± SEM. *p* values were calculated by a one-way analysis of variance followed by Tukey’s test for multiple comparisons. IMP, silicone implants only; IMP + RAD, silicone implants and radiation therapy; IMP + RAD + MSCs, silicone implants, irradiation therapy, and localized administration of human adipose-derived mesenchymal stem cells; TGF-β, transforming growth factor beta; Coll1, collagen type 1; αSMA, alpha-smooth muscle actin; TIMP1, tissue inhibitor of metalloproteinase 1; IL-1β, interleukin-1 beta; TNFα, tumor necrosis factor alpha

**Fig. 3:**
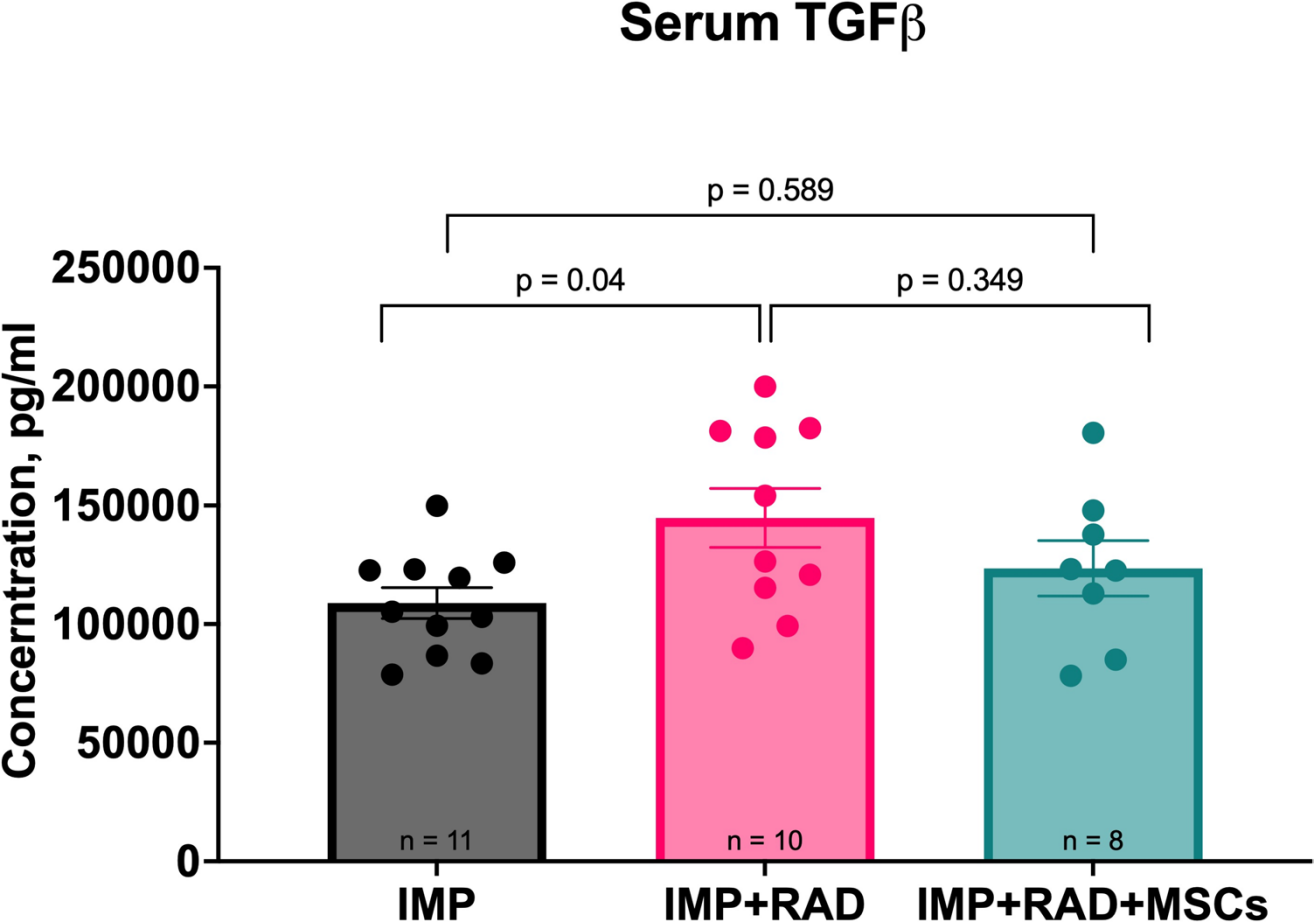
Circulating serum TGF-β concentrations. A mini silicone implant was implanted into mice who then either did not undergo any further treatment (IMP) or were subjected to 10 Gy radiation (IMP + RAD) or radiation as well as localized treatment with human adipose-derived mesenchymal stem cells (IMP + RAD + MSCs). Blood samples were collected 42 days after implantation and serum transforming growth factor beta (TGF-β) concentrations were measured by enzyme-linked immunosorbent assay (ELISA). Data are shown as the mean ± SEM. *p* values were calculated by a one-way analysis of variance, followed by Tukey’s test for multiple comparisons

When compared to the IMP group, mean capsular thickness on day 42 in the IMP + RAD group was significantly increased (233.7 ± 30 μm vs. 483.8 ± 81 μm (*p* = 0.004) (Fig. 1). Radiotherapy also significantly upregulated the expression of pro-fibrotic genes TGF-β, Coll1, αSMA, and TIMP1 in capsular tissue (Fig. 2). Furthermore, pro-inflammatory genes, such as IL-1β and TNFα, were significantly upregulated in the capsular tissues of irradiated mice, suggesting an aggravated local inflammatory response induced by radiation. Finally, a significant systemic increase in serum TGF-β concentrations was observed in irradiated mice compared with IMP controls (144,771 ± 12,417 pg/ml vs.108,879 ± 6513 pg/ml (*p* = 0.04)) (Fig. 3). Taken together, targeted radiotherapy in mice with a subcutaneous silicone mini-implant induced significant CC along with elevated pro-fibrotic and pro-inflammatory responses.

### Human adipose-derived mesenchymal stem cells reduced capsular thickness and the expression of pro-fibrotic and pro-inflammatory genes

When compared to IMP + RAD mice, mice receiving hAD-MSCs treatment showed significantly reduced mean capsular thickness by day 42 after radiation therapy (483.8 ± 81 μm vs. 298.1 ± 25 μm (*p* = 0.045)) (Fig. 1). Notably, hAD-MSC treatment restored capsular thickness to levels similar to those observed in IMP mice (298.1 ± 25 vs. 233.7 ± 30; *p* = 0.624) (Fig. 1).

hAD-MSC treatment significantly downregulated the expression of pro-fibrotic genes TGF-β, Coll1, αSMA, and TIMP1 in capsular tissue (Fig. 2), as well as the expression of the pro-inflammatory genes IL-1β and TNFα. As demonstrated for capsular thickness, the pro-fibrotic gene expression levels returned to baseline in hAD-MSC-treated mice and were similar to those seen in IMP mice (Fig. 2). Circulating TGF-β concentrations were lower in mice treated with hAD-MSCs compared to untreated mice; however, this difference did not reach statistical significance (123,505 ± 11,715 vs. 144,771 ± 12,417; *p* = 0.349), indicating only a non-significant trend (Fig. 3).

**Fig. 4:**
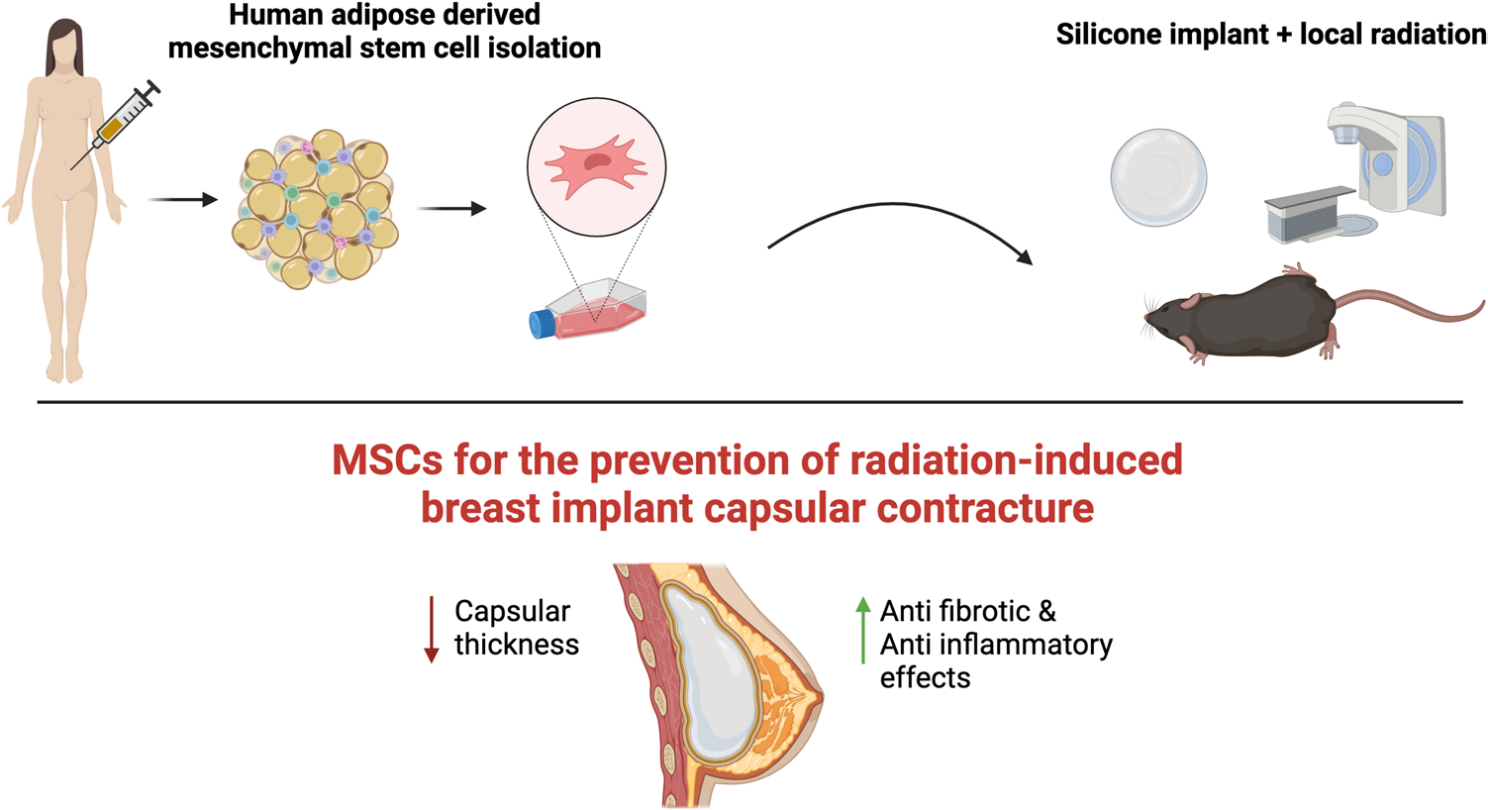
Summary figure. Adipose tissue samples were obtained from patients undergoing liposuction surgery. Human adipose-derived mesenchymal stem cells (hAD-MSCs) were isolated from lipoaspirates and separated from fat by centrifugation. The hAD-MSCs were then seeded into cell dishes and adherent cells were grown for 13 days in growth medium. On day 0, a mini silicone implant device was subcutaneously implanted in 36 female C57Bl/6 wild-type mice. In the radiation groups, the implantation site was immediately subjected to a directed 10 Gy dose of radiation to induce capsular contracture around the mini silicone implants. Animals in the stem-cell group showed significantly reduced capsular thickness and downregulated expression of pro-fibrotic and pro-inflammatory genes 42 days post-radiation

In summary, hAD-MSCs administered immediately after implant insertion and irradiation prevented the increase in capsular thickness and reduced the local pro-fibrotic and pro-inflammatory responses induced by radiotherapy (Fig. 4).

## Discussion

CC is the most common complication of breast augmentation and is likely the primary cause of implant-based breast reconstruction failure, particularly in irradiated patients [3, 6]. Radiation therapy has been shown to induce fibrosis of the capsule and pectoralis muscle, which results in capsular thickening, contracture, and cephalad displacement of the implant. These events are further aggravated by the absence of glandular soft-tissue coverage and by skin flap contracture over the muscle and capsule [3].

The current work demonstrated induction of significant CC following targeted radiotherapy in mice bearing a subcutaneous silicone mini-implant, as demonstrated by a significantly increased implant capsular thickness 42 days following the procedure.

TGF-ß is a key factor in the development of radiation-induced CC and has been termed the “*master switch*” of the TGF-β/Smad axis. Dysregulation of this axis is thought to be central to the development of pathological fibrosis [19, 30]. Indeed, the current work demonstrated a significant upregulation of pro-fibrotic gene (TGF-β, Coll1, αSMA, and TIMP1) and pro-inflammatory gene expression (IL-1β and TNFα) in the capsular tissue of irradiated as compared to control mice. Katzel et al. showed that reduced TGF-β signaling in Smad3 knockout mice eliminated nearly all the signs of CC. A parallel histological analysis showed that blocking TGF-β signaling by Smad3 also significantly reduced implant capsule thickness [19].

In a recent study, Sutthiwanjampa et al. found that hAD-MSCs successfully suppressed fibrotic and inflammatory responses around breast implants in rats, as measured by capsule thickness, collagen density, and myofibroblast and fibroblast counts [33].

In their evaluation of the effect of mouse adipose-derived mesenchymal stem cells (mAD-MSCs) around customized silicone implants on CC, Park et al. measured reduced capsule thickness, decreased myofibroblast, and macrophage counts in the capsule and decreased mRNA levels of fibrogenic genes within the peri-implant tissue [34].

Diehm et al. developed a novel technique for cell-assisted hybrid breast reconstruction (CA-HBR) in a rat model, which had a lower rate of CC. Stem-cell-enriched fat grafting significantly reduced the thickness and collagen density of the capsules and reduced collagen I and TGF-β levels in the capsule tissue [37]. These findings verified the contribution of adipose-derived mesenchymal stem cells to prevention of CC after breast implantation.

However, none of the above models [33, 34, 37] applied any factors to induce CC. The current work demonstrated induction of CC by irradiation which better resembled that of clinical practice. In the present study, hAD-MSC treatment significantly reduced capsular thickness (*p* < 0.05), and significantly downregulated the expression of pro-fibrotic and pro-inflammatory genes in the capsular tissue. Notably, hAD-MSC treatment restored capsular thickness and gene expression to levels similar to those seen in mice with implants who did not receive any radiation at all. Although circulating TGF-β levels were reduced in hAD-MSC-treated animals, this difference did not reach statistical significance, suggesting only a non-significant trend that warrants further investigation.

While the precise mechanisms were not explored in this study, the beneficial effects of hAD-MSCs may be mediated through paracrine signaling, including secretion of extracellular vesicles, anti-fibrotic cytokines, and immunomodulatory molecules. These may act to reduce myofibroblast differentiation, inhibit inflammatory pathways, and limit extracellular matrix deposition. Future studies will be needed to elucidate these pathways.

This work had several limitations. The data were acquired using a mouse model, which may not fully represent human responses. In addition, it utilized customized silicone implants rather than implants specifically designed for human applications.

We acknowledge concerns regarding the use of immunocompetent C57Bl/6 mice for hAD-MSC studies. However, literature supports the validity of this model, demonstrating that hAD-MSCs can survive and function in xenogeneic environments due to their low immunogenicity and immunomodulatory properties [38–40].

Studies have shown that hAD-MSCs effectively reduce inflammatory responses in such models, [33, 41] making this approach suitable for evaluating their potential in capsular contracture prevention.

Furthermore, irradiation and hAD-MSCs were administered within the same anesthetic procedure, thus not fully replicating clinical practice. Additionally, the 42-day follow-up period may have been too short to capture long-term outcomes or CC recurrence; longer term investigations will be necessary. Finally, the potential long-term safety and adverse effects of stem cells warrant further investigation.

## Conclusion

Targeted radiotherapy in implanted mice induced significant CC along with elevated pro-fibrotic and pro-inflammatory responses. This effect was significantly ameliorated by introduction of hAD-MSCs, as shown by prevention of radiation-induced increases in capsular thickness and in local pro-fibrotic and pro-inflammatory responses. The novel animal model used here demonstrated the therapeutic potential of hAD-MSCs in the prevention of breast implant CC.

## Data Availability

The datasets used and/or analyzed during the current study are available from the corresponding author on reasonable request. Data supporting the results reported in the paper are archived in Zenodo 10.5281/zenodo.12788885.

## Abbreviations

CC: Capsular contracture
hAD-MSCs: Human adipose-derived mesenchymal stem cells
TGF-β: Transforming growth factor beta
PMRT: Postmastectomy radiation therapy
PCR: Real-time polymerase chain reaction
ELISA: Enzyme-linked immunosorbent assay
Coll1: Collagen type 1
αSMA: Alpha-smooth muscle actin
TIMP1: Tissue inhibitor of metalloproteinase 1
IL-1β: Interleukin-1 beta
TNFα: Tumor necrosis factor alpha
GAPDH: Glyceraldehyde 3-phosphate dehydrogenase

## References

[1] Susini P, Nisi G, Pierazzi DM, et al. (2023) Advances on capsular contracture - prevention and management strategies: a narrative review of the literature. *Plast Reconstr Surg Glob Open*. 11(6). 10.1097/GOX.0000000000005034 10.1097/GOX.0000000000005034 PMC10256414 37305202

[2] Wagner DS, Mirhaidari SJ. (2021) Capsulectomy, implant exchange, and placement of acellular dermal matrix is effective in treating capsular contracture in breast augmentation patients. *Aesthet Surg J*. 41(3). 10.1093/asj/sjz358 10.1093/asj/sjz358 31826242

[3] Haran O, Bracha G, Tiosano A et al (2020) Postirradiation capsular contracture in implant-based breast reconstruction: management and outcome. Plast Reconstr Surg Published online. 10.1097/PRS.0000000000007453 10.1097/PRS.0000000000007453 33002986

[4] Hammond JB, Kosiorek HE, Cronin PA, et al. (2021) Capsular contracture in the modern era: a multidisciplinary look at the incidence and risk factors after mastectomy and implant-based breast reconstruction. *Am J Surg*. 221(5). 10.1016/j.amjsurg.2020.09.020 10.1016/j.amjsurg.2020.09.020 32988607

[5] Larsen A, Rasmussen LE, Rasmussen LF, et al. (2021) Histological analyses of capsular contracture and associated risk factors: a systematic review. *Aesthetic Plast Surg*. 45(6). 10.1007/s00266-021-02473-3 10.1007/s00266-021-02473-3 34312696

[6] Boyd CJ, Chiodo M V, Lisiecki JL, et al. Plastic and reconstructive surgery advance online article systematic review of capsular contracture management following breast augmentation: an update. 10.1097/PRS.0000000000010358 10.1097/PRS.0000000000010358 36877620

[7] Venkataram A, Lahar N, Adams WP. (2023) Enhancing patient outcomes in aesthetic breast implant procedures using proven antimicrobial breast pocket irrigations: a 20-year follow-up. *Aesthet Surg J*. 43(1). 10.1093/asj/sjac238 10.1093/asj/sjac238 36039664

[8] Hölmich LR, Breiting VB, Fryzek JP, et al. (2007) Long-term cosmetic outcome after breast implantation. *Ann Plast Surg*. 59(6). 10.1097/SAP.0b013e31803c7c78 10.1097/SAP.0b013e31803c7c78 18046137

[9] Handel N, Cordray T, Gutierrez J, Jensen JA. (2006) A long-term study of outcomes, complications, and patient satisfaction with breast implants. *Plast Reconstr Surg*. 117(3). 10.1097/01.prs.0000201457.00772.1d 10.1097/01.prs.0000201457.00772.1d 16525261

[10] Araco A, Caruso R, Araco F, Overton J, Gravante G. (2009) Capsular contractures: a systematic review. *Plast Reconstr Surg*. 124(6). 10.1097/PRS.0b013e3181bf7f26 10.1097/PRS.0b013e3181bf7f26 19952637

[11] Marques M, Brown SA, Oliveira I, et al. (2010) Long-term follow-up of breast capsule contracture rates in cosmetic and reconstructive cases. *Plast Reconstr Surg*. 126(3). 10.1097/PRS.0b013e3181e5f7bf 10.1097/PRS.0b013e3181e5f7bf 20463624

[12] Cordeiro PG, Albornoz CR, McCormick B, Hu Q, Van Zee K. (2014) The impact of postmastectomy radiotherapy on two-stage implant breast reconstruction: an analysis of long-term surgical outcomes, aesthetic results, and satisfaction over 13 years. *Plast Reconstr Surg*. 134(4). 10.1097/PRS.0000000000000523 10.1097/PRS.0000000000000523 25357021

[13] Ricci JA, Epstein S, Momoh AO, Lin SJ, Singhal D, Lee BT. (2017) A meta-analysis of implant-based breast reconstruction and timing of adjuvant radiation therapy. *Journal of Surgical Research*. 218. 10.1016/j.jss.2017.05.072 10.1016/j.jss.2017.05.072 28985836

[14] Rosato RM, Dowden RV. (1994) Radiation therapy as a cause of capsular contracture. *Ann Plast Surg*. 32(4). 10.1097/00000637-199404000-00002 10.1097/00000637-199404000-00002 8210149

[15] Gross E, Hannoun-Levi JM, Rouanet P, et al. (2010) Evaluation of immediate breast reconstruction and radiotherapy: factors associated with complications. *Cancer/Radiotherapie*. 14(8). 10.1016/j.canrad.2010.05.004 10.1016/j.canrad.2010.05.004 20674442

[16] Rohrich RJ, Kenkel JM, Adams WP. (1999) Preventing capsular contracture in breast augmentation: in search of the Holy Grail. *Plast Reconstr Surg*. 103(6). 10.1097/00006534-199905000-00033 10.1097/00006534-199905060-00033 10323717

[17] Bachour Y, Verweij SP, Gibbs S, et al. (2018) The aetiopathogenesis of capsular contracture: a systematic review of the literature. *Journal of Plastic, Reconstructive and Aesthetic Surgery*. 71(3). 10.1016/j.bjps.2017.12.002 10.1016/j.bjps.2017.12.002 29301730

[18] Luvsannyam E, Patel D, Hassan Z, Nukala S, Somagutta MR, Hamid P (2020) Overview of risk factors and prevention of capsular contracture following implant-based breast reconstruction and cosmetic surgery: a systematic review. Cureus Published online. 10.7759/cureus.10341 10.7759/cureus.10341 PMC7549852 33062465

[19] Katzel EB, Koltz PF, Tierney R, et al. (2011) The impact of Smad3 loss of function on TGF-β signaling and radiation-induced capsular contracture. *Plast Reconstr Surg*. 127(6). 10.1097/PRS.0b013e3182131bea 10.1097/PRS.0b013e3182131bea 21617460

[20] Graf R, Ascenço ASK, Freitas RDS, et al. (2015) Prevention of capsular contracture using leukotriene antagonists. *Plast Reconstr Surg*. 136(5). 10.1097/PRS.0000000000001683 10.1097/PRS.0000000000001683 26505715

[21] Gancedo M, Ruiz-Corro L, Salazar-Montes A, Rincón AR, Armendáriz-Borunda J. (2008) Pirfenidone prevents capsular contracture after mammary implantation. *Aesthetic Plast Surg*. 32(1). 10.1007/s00266-007-9051-4 10.1007/s00266-007-9051-4 17968613

[22] Caffee HH. (2002) Capsule injection for the prevention of contracture. *Plast Reconstr Surg*. 110(5). 10.1097/01.prs.0000025628.86133.ee 10.1097/01.PRS.0000025628.86133.EE 12360077

[23] Planas J, Migliano E, Wagenfuhr J, Castillo S. (1997) External ultrasonic treatment of capsular contractures in breast implants. *Aesthetic Plast Surg*. 21(6). 10.1007/s002669900143 10.1007/s002669900143 9354599

[24] Johnson JD, Glat PM, Scarlett WL. (2015) Low-level laser therapy: an alternative treatment for capsular contraction. *The American Journal of Cosmetic Surgery*. 32(1). 10.5992/ajcs-d-14-00036.1

[25] Diehm YF, Hirche C, Berger MR, et al. (2019) The collagenase of the bacterium clostridium histolyticum in the treatment of irradiation-induced capsular contracture. *Aesthetic Plast Surg*. 43(3). 10.1007/s00266-018-1267-y 10.1007/s00266-018-1267-y 30456640

[26] Lombardo GAG, Tamburino S, Magano K et al (2020) The effect of omega-3 fatty acids on capsular tissue around the breast implants. Plast Reconstr Surg Published online. 10.1097/PRS.0000000000006553 10.1097/PRS.0000000000006553 32097310

[27] Wan D, Rohrich RJ. (2016) Revisiting the management of capsular contracture in breast augmentation: a systematic review. *Plast Reconstr Surg*. 137(3). 10.1097/01.prs.0000480095.23356.ae 10.1097/01.prs.0000480095.23356.ae 26910663

[28] Evans GRD, Widgerow AD. (2020) Stem cells and tissue engineering in plastic surgery: an update. *Plast Aesthet Res*. 7. 10.20517/2347-9264.2019.53

[29] Gimble JM, Katz AJ, Bunnell BA. (2007) Adipose-derived stem cells for regenerative medicine. *Circ Res*. 100(9). 10.1161/01.RES.0000265074.83288.09 10.1161/01.RES.0000265074.83288.09 PMC5679280 17495232

[30] Li ZJ, Wang LQ, Li YZ, et al. (2021) Application of adipose-derived stem cells in treating fibrosis. *World J Stem Cells*. 13(11). 10.4252/wjsc.v13.i11.1747 10.4252/wjsc.v13.i11.1747 PMC8641015 34909121

[31] Shukla L, Yuan Y, Shayan R, Greening DW, Karnezis T. (2020) Fat therapeutics: the clinical capacity of adipose-derived stem cells and exosomes for human disease and tissue regeneration. *Front Pharmacol*. 11. 10.3389/fphar.2020.00158 10.3389/fphar.2020.00158 PMC7062679 32194404

[32] Vieira VJ, D’Acampora A, Neves FS, et al. (2016) Capsular contracture in silicone breast implants: insights from rat models. *An Acad Bras Cienc*. 88(3). 10.1590/0001-3765201620150874 10.1590/0001-3765201620150874 27627068

[33] Sutthiwanjampa C, Shin BH, Ryu NE, Kang SH, Heo CY, Park H (2021) Assessment of human adipose-derived stem cell on surface-modified silicone implant to reduce capsular contracture formation. Bioeng Transl Med. 10.1002/btm2.10260 35111952 10.1002/btm2.10260 PMC8780897

[34] Park BY, Wu D, Kwon KR, et al. (2023) Implantation and tracing of green fluorescent protein-expressing adipose-derived stem cells in peri-implant capsular fibrosis. *Stem Cell Res Ther*. 14(1). 10.1186/S13287-023-03248-0 10.1186/s13287-023-03248-0 PMC9906918 36750973

[35] Adams WP, Haydon MS, Raniere J et al (2006) A rabbit model for capsular contracture: development and clinical implications. Plast Reconstr Surg 117(4):1214–1219 16582789 10.1097/01.prs.0000208306.79104.18

[36] Katzel EB, Koltz PF, Tierney R et al (2010) A novel animal model for studying silicone gel-related capsular contracture. Plast Reconstr Surg 126(5):1483–1491 21042104 10.1097/PRS.0b013e3181ef8b8e

[37] Diehm YF, Thomé J, Will P et al (2023) Stem cell-enriched hybrid breast reconstruction reduces risk for capsular contracture in a hybrid breast model. Plast Reconstr Surg 152(3):572–580 36735813 10.1097/PRS.0000000000010260

[38] Ankrum JA et al (2014) Mesenchymal stem cells: immune evasive, not immune privileged. Nature Biotechnology 323(2014):252–60 10.1038/nbt.2816 PMC4320647 24561556

[39] Uccelli A et al (2008) Mesenchymal stem cells in health and disease. Nat Rev. Immunol 8,9(2008):726–36 19172693 10.1038/nri2395

[40] Prockop, Darwin J et al (2017) Data against a common assumption: xenogeneic mouse models can be used to assay suppression of immunity by human MSCs. Mol Ther J Am Soc Gene Ther 258:1748–1756 10.1016/j.ymthe.2017.06.004 PMC5542802 28647464

[41] Jaber, Hala et al (2021) The therapeutic effects of adipose-derived mesenchymal stem cells on obesity and its associated diseases in diet-induced obese mice. Scientific Rep 111:6291 10.1038/s41598-021-85917-9 PMC7973738 33737713

